# F172. INDIVIDUAL PREDICTION OF RISK IN ADOLESCENT OFFSPRING OF PARENTS WITH SCHIZOPHRENIA OR BIPOLAR DISORDER: A MACHINE LEARNING NEUROIMAGING STUDY WITH A CROSS-STAGE VALIDATION

**DOI:** 10.1093/schbul/sby017.703

**Published:** 2018-04-01

**Authors:** Hugo Schnack, Julia Binnewies, Nikita Setiaman, Woutje Berdowski, Neeltje van Haren, René Kahn, Manon Hillegers

**Affiliations:** UMC Utrecht

## Abstract

**Background:**

Schizophrenia (SZ) and bipolar disorder (BD) are severe psychiatric disorders that are not easily distinguishable based on clinical measures. Offspring of patients with SZ or BD have a tenfold increased risk of developing the disorder as well as an increased risk for other severe mental disorders. Reliable identification of these subjects might allow for early recognition and intervention, which have been shown to be beneficial for treatment outcome and may even prevent transition to illness. Based on abundant evidence that SZ and BD are associated with structural brain abnormalities, we investigated whether MRI brain-scans can be used to detect individual risk of developing SZ or BD in adolescents.

**Methods:**

Structural MRI brain-scans were acquired in adolescent offspring (8–19 year) of parents with schizophrenia (oSZ;N=50), bipolar disorder (oBD;N=82), and without a mood or psychotic DSM-IV disorder (oHC;N=53), as part of the Dutch Bipolar and Schizophrenia Offspring Study (DBSOS). Support vector machine (SVM) models were trained on the gray matter tissue density maps to predict to which offspring class (oHC/oBD/oSZ) an individual belonged. Prediction accuracy was assessed using cross-validation. To validate our prediction models, we applied them to the tissue maps from subjects from a sample of unrelated HC/BD/SZ adults. Secondly, validated prediction models built from the adult subjects’ MRI scans were applied to the tissue maps of the adolescents to predict illness class (HC/BD/SZ).

**Results:**

The offspring-based model separated oHC/oSZ individuals with 77% accuracy (p<0.001), oHC/oBD with 68% accuracy (p<0.001), and oBD/oSZ with 64% accuracy (p<0.01). The adult-based models could separate the patients’ offspring from the healthy offspring with 66–70% accuracy, but oBD from oSZ with lower accuracy (59%). In addition, the offspring models could separate adult patients from control subjects with comparable accuracy (66–68%) and separate the two patient groups with moderate accuracy (69%).

**Discussion:**

The familial high-risk adolescents could be separated from controls with moderate to high accuracy (up to 77%), based on their MRI-scans. Moreover, the brain tissue patterns based on risk (adolescents) or illness (adults) were able to predict (risk) class in the other stage group. These results show (1) that high-risk individuals already show brain abnormalities, and (2) display similarities with abnormalities in ill adults, and (3) which can be used to detect (risk of) the disorder at the individual level. This suggests that MRI-scans, after further improvement and independent validation, may be of added value in the risk profiling of BD and SZ.

